# Treatment of Recently Exposed Nerves with Nitric Acid—with Cases

**Published:** 1859-08

**Authors:** J. Taft


### TREATMENT OF RECENTLY EXPOSED NERVES WITH NITRIC ACID—WITH CASES.

BY J. TAFT.

AT the meeting of the American Convention, in Cincinnati, in August last, the treatment of recently exposed nerves, with nitric acid, was first suggested in our hearing, if memory is correct, by Dr. Wright of Pittsburgh. The idea struck almost every one present, as somewhat novel in character. Dr. W. represented his success in this treatment as almost universal. Upon the representations made, I was induced to try it, and the result has been success beyond all anticipation.—The treatment seems to be well adapted where the nerve is exposed by excavation, or but recently exposed in any way. Patients having a good constitution, with strong vitality, and of good recuperative powers, would be preferable, though it may answer well, in cases of feeble vitality,

Wounding the pulp in excavation is a matter of no consequence, it is probably quite as well to be punctured, and a little blood taken from it.

After the cavity of decay is entirely excavated and ready for filling, it should be wiped out with cotton, then take a pine stick and dress it down till the end is about the same size as the orifice at which the pulp is exposed, then dip it into chemically pure nitric acid, then touch the pulp at the point of exposure, with the end of the stick thus charged, retain it there from one-third to a whole minute, when the pulp at the point of exposure will be found cauterized and blackened; the operation of filling may then at once be performed, ordinarily without any intervening subtance between the gold and cauterized part; this, however, in some cases would be indicated, especially if the pulp was exposed at comparatively a large orifice. Introducing the filling if properly performed, gives no pain.

The following cases pretty fairly represent the treatment and success of my practice in this particular.

CASE 1ST.—June 26th, 1859, Mrs. E. aged 24 years, strumous diathesis. Had several teeth filled. The left central superior incisor had a large cavity in the central proximal surface; after excavating the pulp was exposed at a small point; it was touched with a sharp instrument and bled slightly. The tooth had not ached, and the pulp was healthy. Touched it with nitric acid till the point of exposure was blackened, then dried out and filled as usual, without causing any pain, had no intervening substance.

CASE 2ND. — Same patient, same time. The superior cuspid of the left side was largely decayed, on the anterior proximal surface. The cavity about two months before, had been *stuffed*, by one who did not know what to do with it, the pulp being slightly exposed, was very sensitive, and had been in that condition all the while since it was thus *stuffed*. Removed the loose gold from the cavity, and found decay going on rapidly, and the pulp exposed at a considerable orifice, and in quite an irritable condition, it bled freely at a touch. Cleaned and formed the cavity, touched the pulp at the point of exposure with nitric acid, till it was blackened, after which there was some pain, applied creosote for a few moments, by which the pain was wholly subdued: then filled the tooth in the usual manner without causing pain, used no intervening substance. This is the most unfavorable case in which I have employed the nitric acid treatment. Two days afterward saw the teeth, and they were doing well, not the slightest uneasiness had been experienced by the patient.

CASE 3RD.—July 9th, 1859, Mrs. L., aged 22 years, feeble constitution. Had the first left superior bicuspid largely decayed on the anterior proximal surface. Pulp was exposed, but in a healthy condition. Excavated the cavity without wounding the pulp, touched it with nitric acid, and filled as usual, without pain.

June 12th.—Since this tooth was filled there has been some pain, of neuralgic character. The case is a doubtful one.

In many cases the application of the acid produces no pain whatever; in other a sharp twinge is experienced at first touch. In a healthy pulp, there is little or no pain, while those in a state of irritation, are usually somewhat painful under the application.

